# The tendency to stop collecting information is linked to illusions of causality

**DOI:** 10.1038/s41598-021-82075-w

**Published:** 2021-02-16

**Authors:** María Manuela Moreno-Fernández, Fernando Blanco, Helena Matute

**Affiliations:** 1 Department of Developmental and Educational Psychology, Faculty of Psychology, University of Granada, Granada, Spain; 2 Department of Methods and Experimental Psychology, Faculty of Psychology and Education, University of Deusto, Bilbao, Spain; 3 Department of Social Psychology, Faculty of Psychology, University of Granada, Granada, Spain

**Keywords:** Psychology, Human behaviour

## Abstract

Previous research proposed that cognitive biases contribute to produce and maintain the symptoms exhibited by deluded patients. Specifically, the tendency to jump to conclusions (i.e., to stop collecting evidence soon before making a decision) has been claimed to contribute to delusion formation. Additionally, deluded patients show an abnormal understanding of cause-effect relationships, often leading to causal illusions (i.e., the belief that two events are causally connected, when they are not). Both types of bias appear in psychotic disorders, but also in healthy individuals. In two studies, we test the hypothesis that the two biases (jumping to conclusions and causal illusions) appear in the general population and correlate with each other. The rationale is based on current theories of associative learning that explain causal illusions as the result of a learning bias that tends to wear off as additional information is incorporated. We propose that participants with higher tendency to jump to conclusions will stop collecting information sooner in a causal learning study than those participants with lower tendency to jump to conclusions, which means that the former will not reach the learning asymptote, leading to biased judgments. The studies provide evidence in favour that the two biases are correlated but suggest that the proposed mechanism is not responsible for this association.

## The tendency to stop collecting information is linked to illusions of causality

In recent years, there has been a growing interest in exploring biased cognition as a potential mechanism underlying dysfunctional behaviours displayed by psychotic patients^1–5^. Empirical studies from this perspective have yielded evidence for cognitive biases as contributors to the onset and maintenance of symptoms in deluded patients^6–11^. For example, one cognitive bias that has been extensively explored in deluded patients is the tendency to Jump to Conclusions (JtC)^7,12^. Individuals with tendency to jump to conclusions typically use a reduced amount of evidence to make their decisions under uncertainty. Generally, people subject to this bias stop collecting information about a problem at earlier stages of the information search process, compared to people who do not display the bias, or who do it at a lower extent. This eventually leads to hasty decisions based on a limited amount of evidence, which may in turn contribute to delusion formation^12^.

Nevertheless, cognitive biases such as JtC are not specific features of clinical states. Rather, they have been found in the general population^13,14^ and often in association with subclinical manifestations of psychosis^9,11,15^. For example, the tendency to jump to conclusions (JtC) in healthy individuals has been found linked to the presence of mistaken inferences, such as delusional ideation and delusion-proneness^16,17^, paranormal beliefs^18,19^, or confidence in paranoid thoughts and experiencing perceptual anomalies^13^. This evidence has been put forward to suggest that cognitive biases may be considered as a transdiagnostic mechanism involved in predisposing an individual to develop and/or maintaining a disorder^20,21^.

Furthermore, the proposal that cognitive biases are not restricted to pathological states and that they often appear in healthy individuals is reinforced by research showing that even experts can fall prey of biases routinely. For instance, forensic professionals can be biased by irrelevant contextual information^22,23^ or by the side who requested their judgment (“adversarial allegiance”)^24^. Similarly, the judgment of scientists can also be affected by base-rates, expectancies, and other (in principle) irrelevant pieces of information when drawing conclusions from data^25^. This aligns with theoretical views that understand cognitive biases as the result of general mechanisms that operate in all individuals^26^, such as emotions, social influence, or even more basic processes such as associative learning^27^. These mechanisms may provide solutions with acceptable success most of the time. However, just as it happens with optical illusions, under certain situations or materials these same mechanisms can lead to biased or erroneous conclusions^28^.

## Abnormal cause–effect relationships and the psychosis continuum: from causal illusions to delusions

Considering cognitive biases as general mechanisms rather than disorder‐specific patterns of impairment^20,21^ fits well with the idea of a continuum between subclinical psychotic experiences and psychotic disorders. From this continuity perspective, symptoms of psychosis such as delusions and hallucinations are not qualitatively different from normal experiences^29–36^. For example, a prominent feature of delusional thinking is the abnormal perception of the relationships between events^37^. Patients with schizophrenia often maintain deviating views on cause-effect relationships^38^, but these deviations can also be detected in the general population, for example, in the form of *causal illusions*^27,39^.

Inferring causal relationships between events is not an easy task because causality is not directly observable^40^. People need to use indirect cues (based on general principles of causation) to assess causal relations between events; for example, causes usually precede their effects (priority principle), and they are usually close to each other in time and space (contiguity principle). An additional condition that has to be met for effective causal estimation is the contingency principle, which implies that the potential cause and its alleged effect must covary with each other^41^.

Previous research has shown that, although people can use the contingency between cause and effect to infer causality^42–47^, under some circumstances, they can easily develop a causal illusion, that is, the belief that there is a causal connection between two events that are actually unrelated (i.e., non-contingent on each other). Causal illusions have been described as cognitive biases that appear in the general population and may underlie many relevant and societal problems, such as prejudice and pseudoscience^27,39,48–51^.

Research on causal illusions has identified some factors that increase the probability of experiencing these false beliefs of causality. One of these factors is the probability with which the cause and the effect are presented. Thus, the higher the probability of the cause, the higher the contingency reported between cause and effect, even in the case in which the actual contingency is null^52–54^. A similar effect has been described when the effect is presented with high frequency^53,55–58^.

Causal illusions are actually very similar to delusions as both refer to beliefs based on incorrect inferences that deviate from reality, but the former could be considered as a “soft version” of delusions, and they can be commonly observed in non-clinical population. In fact, recent research has found that, although causal illusions can be detected in healthy individuals, patients with schizophrenia have significantly greater susceptibility to them^59,60^.

In particular, Balzan et al.^59^ proposed that delusions could result from (or be maintained by) false associations between events for which no association actually exists, a description that overlaps with our definition of causal illusion. Thus, from this perspective, causal illusions may contribute to delusions. For example, as noted by Bazan et al.^59^, the illusion of control, which is a particular type of causal illusion referred to personal control^61^, may have a singular role in grandiose delusions, in which overestimations of personal control may lead patients into believing that they have extraordinary abilities.

We have already discussed on the relevant role of cognitive biases on the development and maintenance of delusions; specifically, we have described JtC as a relevant marker for these pathological beliefs. However, and besides the direct parallel between delusions and delusion-like beliefs such as the illusion of causality, the role of JtC on causal illusions has not been explored yet. Nevertheless, as we will argue next, some theoretical accounts of causal illusions clearly predict a relevant role of the JtC bias on causal illusions.

## The associative account of causal illusions and data gathering effects

A prominent theory to explain causal illusions has been developed from associative learning theories that aim to model human and animal learning. From this perspective, causal beliefs emerge because people learn the associations in their environment, and causal illusions are the result of an incomplete or pre-asymptotic learning experience^39^. According to this view, the formation and strengthening of the associations depend on the general mechanisms of Pavlovian and instrumental learning^62,63^. It has been shown that, under certain circumstances, associative learning models such as the Rescorla and Wagner’s model (1972) predict a temporary overestimation of the link between two events (cause and effect) during the initial stages of training. Then, they predict a subsequent, gradual adjustment to the actual contingency as the training progresses^63,64^. The reason for this pre-asymptotic overestimation of causality is the formation of a spurious association between cause and effect due to the accumulation of trials containing both the cause and the effect, as can be seen in computer-simulation^39^. There is also experimental evidence congruent with this prediction of associative models^45,65–67^.

Thus, it is possible to interpret causal illusions as the result of a spurious association that appears early in the learning session and then disappears. Now, we argue that other cognitive biases that have been connected to certain pathologies could also play a role in this process. Specifically, people with a marked tendency to jump to conclusions will choose to end the training session sooner, and consequently they will expose themselves to reduced training schedules. Since the causal illusion, according to the associative framework, appears at the beginning of the session, those individuals who quit the training stage before reaching the learning asymptote should show stronger illusions. More generally, the tendency to jump to conclusions implies a reduced experience with causes and effects, hence compromising the representativeness and quality of the information that is used for causal inference.

Contingency learning research has provided indirect evidence congruent with this hypothesis. For example, individuals with anomalous beliefs (e.g., paranormal beliefs or superstitions) are more vulnerable to causal illusions^49,68,69^ and these beliefs have also been found associated to the tendency to jump to conclusions^14,18,19^.

Although the link between JtC and delusion proneness has received great attention from researchers^13^, to our knowledge, there are no studies investigating the potential contribution of JtC to contingency learning errors in general population. The present article will try to fill this gap.

## Overview of the studies

In the current research, we examined whether causal illusions were associated with the tendency to jump to conclusions, and whether this association can be explained by the type of learning mechanism proposed by associative models.

**Figure 1:**
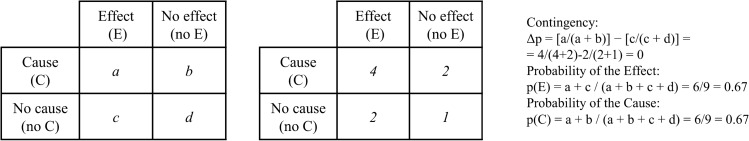
Contingency matrix with the four trial types that could be presented on the contingency learning task. There are four different pieces of information or trials (“a”, “b”, “c”, and “d trial) as a function of whether or not the cause and the effect are present (left panel). From this information, a measure of contingency (Δp)^42^ can be computed as the probability of the effect conditional on the cause occurrence minus the probability of the effect conditional on the cause absence (see example of these computations on the right panel). Positive values indicate a generative causal relationship between the events, and negative values indicate a preventative causal relationship. When both events are not related to each other, the index equals zero and the contingency is null. Causal illusions are usually detected on null contingency settings when the cause and the effect appear with a high probability.

As we will detail later in the Method section, we will use a contingency learning task to measure causal illusions using a set of parameters (null contingency, high probability of the cause and high probability of the effect) known to successfully produce causal illusions^27^. During this task, participants will be asked to gather information to assess whether two events are causally related. Thus, they will be presented with a number of trials (pieces of evidence) in which a potential cause and its alleged effect could be either present or absent (see details on Fig. 1, left Panel). This learning task will be followed by a standard instrument to measure JtC, the Beads task^7,12^ (see details on next section) . We expect that those participants with high tendency to jump to conclusions (as evidenced in the Beads task) will gather a smaller amount of information in the contingency learning task. Then, in line with the associative account for the causal illusion that predicts transient biases, we expect that the greater the amount of information gathered, the more accurate the causal estimation.

### Ethics statement

The Ethical Review Board of the University of Deusto reviewed and approved the methodology reported in this article, and the studies were conducted according to the approved guidelines. Informed consent was obtained from all participants.

## Study 1

### Method

#### Participants

A sample of 100 native English-speakers adults (53 men, 46 women, and one non-binary, *M*_*age*_ = 30, *SD* = 9.55) were recruited via Prolific Academic^70^, and were compensated for their participation with £1.25 (£5.04 per hour). This sample size allows to detect an effect of *r* = 0.24 or smaller with 80% power, as revealed by a sensitivity analyses^71^ conducted in G*Power^72^. We did not stop the data collection before reaching the planned sample (*n* = 100). The participation was offered only to those applicants in Prolific Academic’s pool with English as their first language (to ensure that instructions were correctly understood) and who had not taken part in previous studies carried out in Prolific Academic by our research team.

### Instruments and apparatus

To assess causal illusions and Jumping to Conclusions, we used two computerized adaptations of widely used tasks: the *contingency learning task*^53,55,73^ and the *Beads task*^7,12^, respectively. These two adaptations were presented as a web application based on World Wide Web Consortium (W3C) standards (i.e., HTML, CSS, and JavaScript). Participants were required to use a desktop computer and the Google Chrome browser to ensure compatibility, and they could quit the study at any moment by closing the browser window. No personal information (i.e., name, IP address, e-mail) was collected, neither did we use cookies or other software to covertly obtain information from the participants. A demo of this task can be downloaded from the Open Science Framework (OSF).

### Procedure and design

The initial instructions asked participants not to jot down notes during the study. Then, the contingency learning task was presented.

#### Contingency learning task

Participants were required to imagine that they were doctors working at a research laboratory, and that they had to find out whether or not a medicine (i.e., Batatrim) was effective for healing the crises of a rare and dangerous disease (i.e., Lindsay Syndrome). See detailed instructions on Supplementary Appendix A. Note that the scenario is fictional, as neither the medicine nor the disease actually exist.

**Figure 2:**
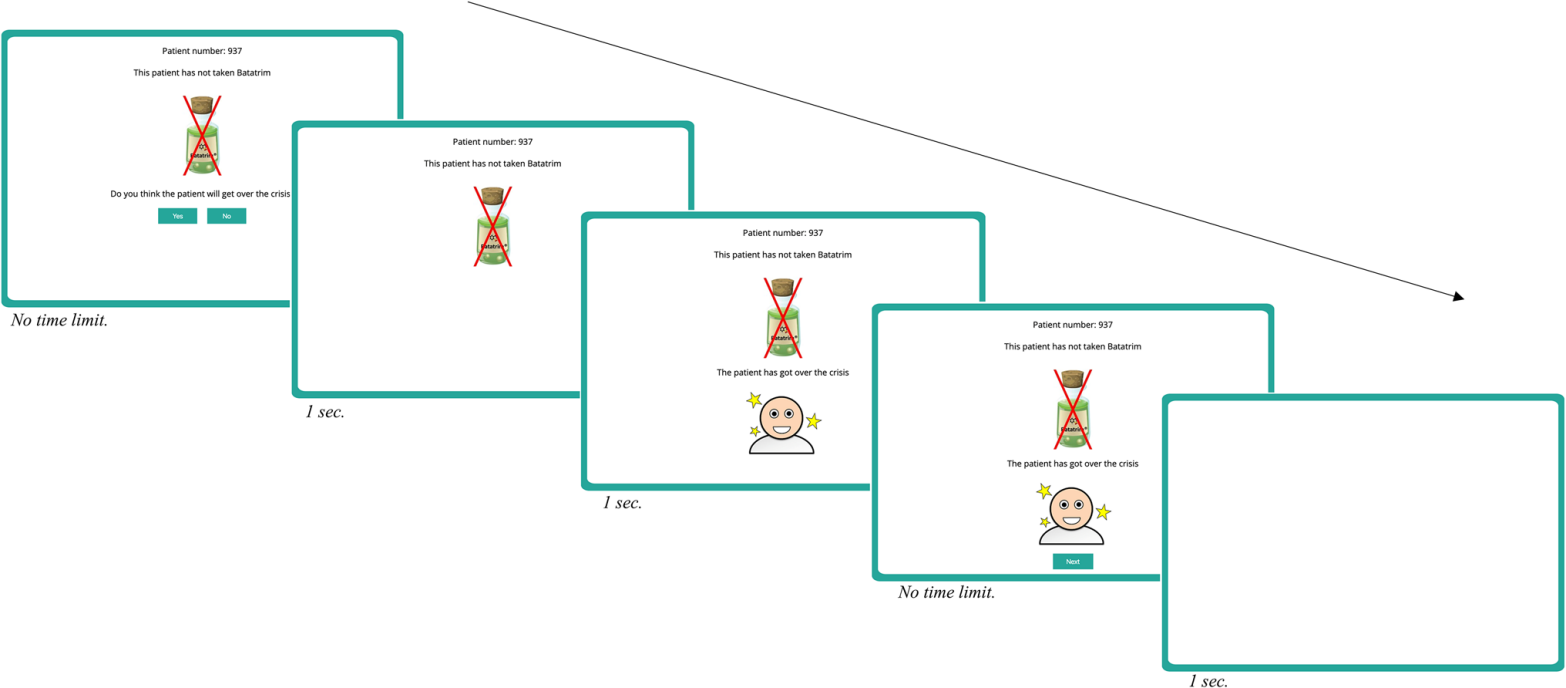
Schematic procedure of the contingency learning task used in Study 1 (this figure has been made up by combining actual screenshots from the original task).

After reading the instructions, participants were exposed to a series of blocks of nine trials. On each trial, an idealized medical record of a patient suffering from a crisis of Lindsay Syndrome was presented (see Fig. 2). At the top of the screen, the words “Patient number:” followed by a random number were presented to ensure that participants perceived each medical record as an independent case. This information was accompanied by a figure depicting the treatment followed by that particular patient (i.e., either treated with Batatrim—a drawing of a bottle labelled with a molecule symbol and the name “Batatrim”—or not treated with Batatrim—a drawing of a bottle labelled with a molecule symbol and the name “Batatrim” crossed out in red—), and a predictive question asking participants to guess whether the patient would get over the crisis or not. Note that participants could not predict the outcome (overcoming the crisis or not) for each patient, since there were no visual cues in the medical record that could help with the prediction. These trial-by-trial predictive questions were included to maintain the participants’ attention during the task, but the responses were not used for the analysis. Once the prediction was made, the question disappeared and a cartoon representing the patient (either recovered or ill) was presented together with a written description (“The patient has [has not] got over the crisis”). Therefore, each trial presented one of the four types of trial described in Fig. 1.

Finally, a button labelled “Next” appeared, allowing to proceed to the next case. Once participants clicked on the button, the screen was cleared, and a new trial was presented. There was no time-limit for advancing through the task (participants advanced at their own pace).

After the ninth trial of each block, participants were required to choose between (a) continuing checking more records, or (b) proceeding to rate the effectiveness of Batatrim. Those participants who decided to inspect more records were presented with a new series of nine cases (i.e., trials) in random order, following the procedure previously described, whereas those who decided to answer were presented with the following text to confirm their choice: *“We still have medical records of patients with Lindsay Syndrome which we haven’t shown to you. You can see some more records or let us know if Batatrim is effective to heal the Lindsay Syndrome. If you need to, we recommend you to see more records”.* This text was presented with two buttons labelled with the words “I want to see more patient records” and “I want to answer”. Then, participants who confirmed that they were ready to quit the training stage were required to use a 101-point scale to rate the effectiveness of the medicine, that is, to judge the causal relation between taking the medicine and overcoming the crisis (judgement of causality). This scale was labelled at three points: 0, the left end (Non-effective); 50, the middle point (Quite effective); and 100, the right end (Totally effective).

The above described contingency learning task allowed participants to explore a maximum of ten blocks of nine trials each, that is, they could explore a total of 9, 18, 27, 36, 45, 54, 63, 72, 81, or 90 medical records. All blocks contained the same information: 4 “a” trials, 2 “b” trials, 2 “c” trials, and 1 “d” trial (following the notation in Fig. 1), presented in random order. This ensured that, independently of the number of training blocks that they saw, all participants were exposed to a null contingency setting (Δp = 0) with a high probability of the effect (i.e., high probability of healings) and a high probability of the cause (i.e., high probability of being treated with Batatrim). Both probabilities were fixed to 0.67, that is, the training could vary only in its length, but not in any other relevant parameter (see an example with one block of trials in Fig. 1, right panel). We will use the number of training blocks as a measure of training length (i.e., the amount of information gathered) and judgements of causality as a measure of causal illusions (note that contingency between the cause and the effect was fixed to zero and that an accurate value for the causal judgement should be zero). We expected that people exposed to shorter training schedules (those who gathered less information) would display stronger illusions, according to associative learning models.

#### Beads task

Our version of the Beads task was presented immediately after the contingency learning task. The initial instructions reminded participants not to jot down notes and described the rules for this second phase (see detailed instructions in Supplementary Appendix B). Participants were informed that one of two containers filled with 100 beads had been poured into a box. Both containers had red and blue beads, but in different proportions. Thus, one container had 60 red beads and 40 blue beads, whereas the other one had the inverse proportion, 40 red beads and 60 blue beads^8^. The participants’ goal on this second task was to find out which one of the two containers (the one with more red beads, or the one with more blue beads) had been poured into the box.

**Figure 3:**
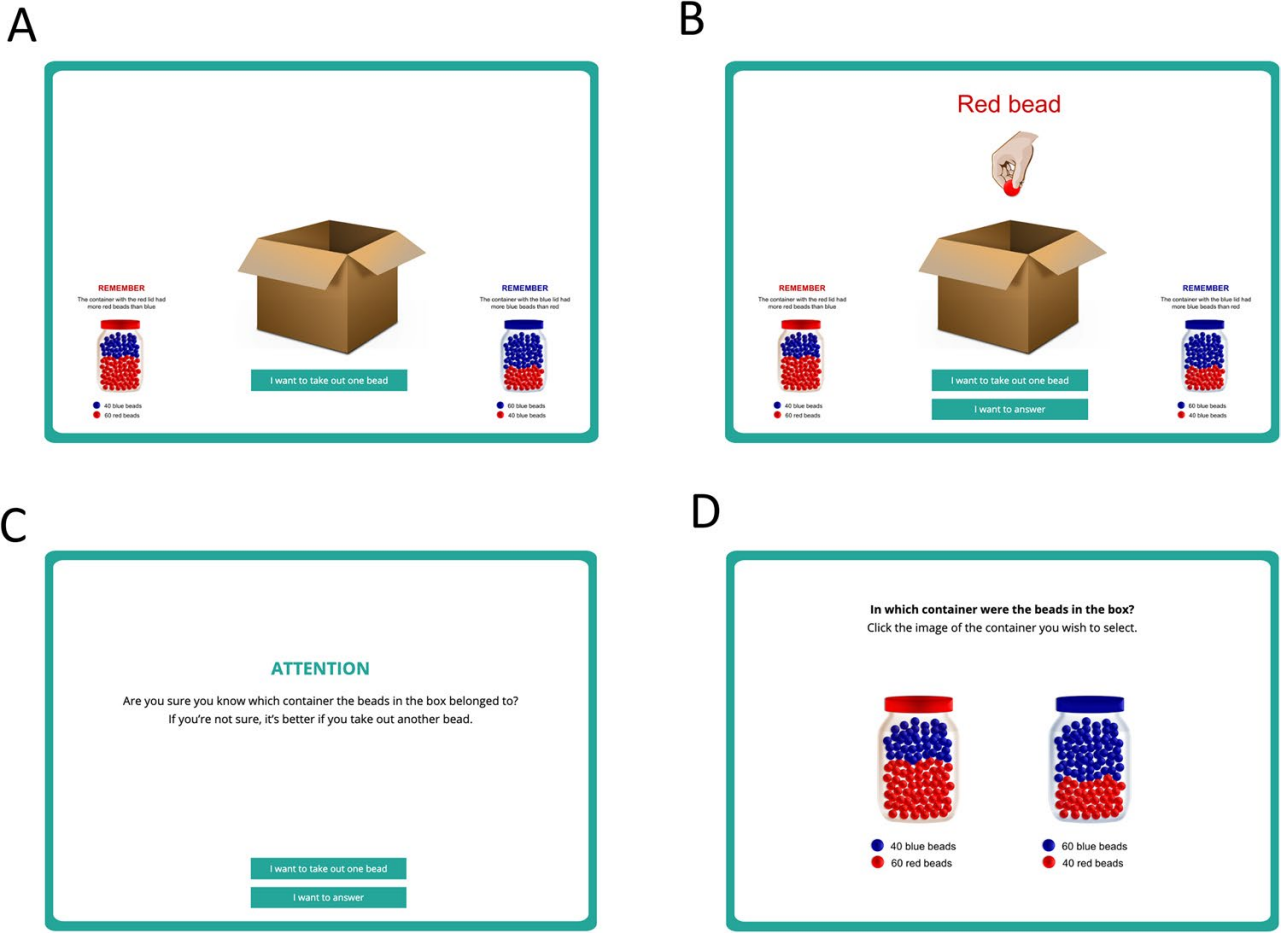
Beads task. Panel **(A)** displays how information was presented on the first trial. Note that participants are forced to take the first bead. The remaining 49 trials allow participants to take an additional bead or to give an answer, see Panel **(B)**. Panel **(C)** shows the confirmation screen. Finally, panel **(D)** displays how the final choice between containers was requested.

To answer this question, they could take beads from the box. The instructions explained that beads should be taken one by one, and put back inside the box before the next extraction. This ensures that the red and blue bead proportions are held constant (i.e., the extraction is random with replacement). After each draw, participants should decide if they wanted to take another bead, or to stop collecting information and report which of the two containers was poured into the box (see Fig. 3 for details). All participants were presented with the same fixed sequence of 50 beads used previously by Ross et al.^14^, which means that participants differed only on the amount of beads tken.

The interpretation of this task is as follows: the fewer beads a participant takes out, the stronger the jumping to conclusions trait is for that participant. We expected that people with stronger jumping to conclusion bias would also be hastier during the contingency learning phase, thereby exploring a reduced amount of evidence, and reporting medicine effectiveness sooner. This in turn, according to associative learning theories, should produce stronger illusions of causality, that is, higher judgements about the effectiveness of Batatrim.

### Results

**Figure 4:**
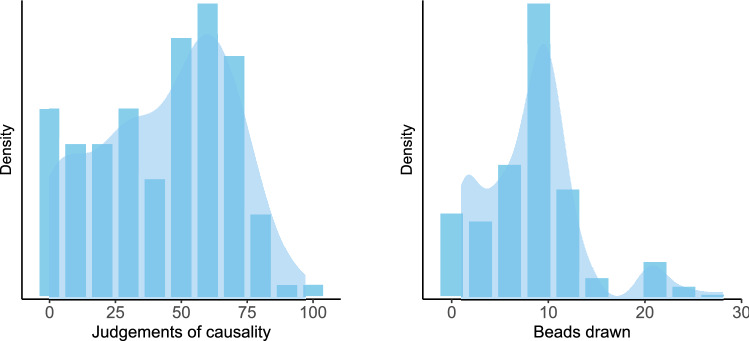
Histograms and density plots for the contingency learning task (left panel) and Beads task (right panel) in Study 1.

Judgements of causality were used as a measure for causal illusions^27^. These judgements varied from 0 to 97 (*M* = 43.28, *SD* = 25.50, see left panel of Fig. 4), that is, although some participants (10 out of 100) noted that there was no relation between using the medicine and recovering from the crisis, most of them overestimated the effectiveness of the medicine, hence displaying an illusion of causality. Previous studies showed similar results^53,74^.

In the Beads task, the number of beads drawn before making a decision ranged between 1 and 28 (*M* = 8.53, *SD* = 5.48; see data distribution on the right panel of Fig. 4). The performance in this task was very close to that reported by Ross et al.^14^, also with general population.

**Figure 5:**
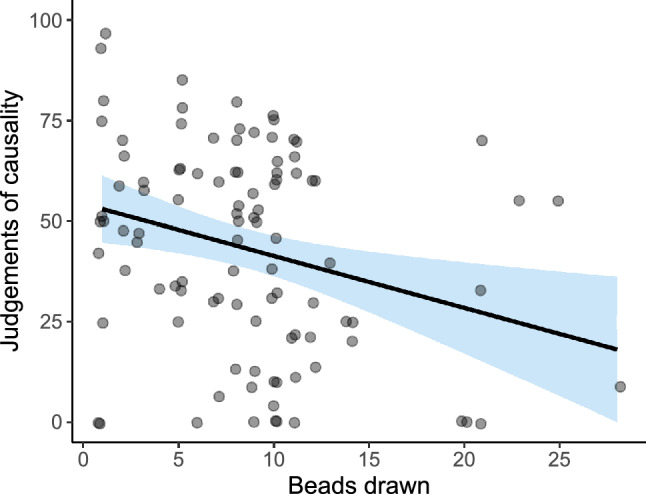
Scatterplot depicting judgements of causality as a function of JtC measure in Study 1. The scatterplot shows causal judgements (vertical axis), and the number of beads drawn in the Beads task (horizontal axis), which assesses the JtC bias. The line fitting the data points displays a negative slope, indicating that those participants who required more information in the Beads task were also more accurate in their causal estimations. We added horizontal jitter to the data points to prevent overplotting.

As expected, we found a significant correlation between the number of beads drawn in the Beads task and causal judgements on the contingency learning task (see Fig. 5). The smaller the number of beads drawn (i.e., the higher the tendency to jump to conclusions), the higher the causal judgements and, therefore, the stronger the illusion of causality (*r* = − 0.28, *p* = 0.005). This was our first prediction, that JtC and causal illusions, which represent cognitive biases that could be measured in the general population, would be correlated with each other.

**Figure 6:**
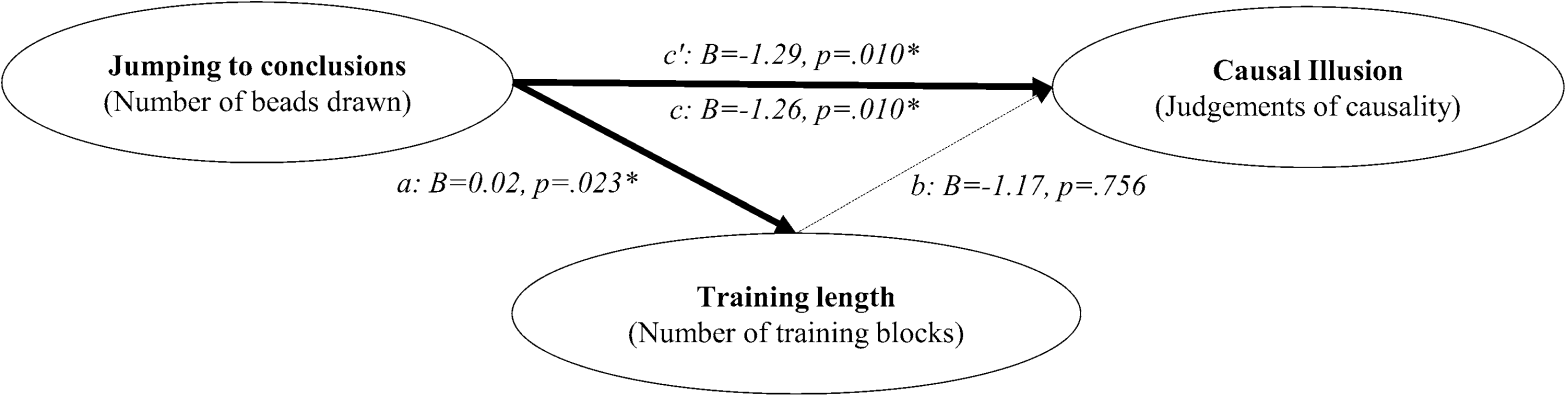
Path model in Study 1. The letters a, b, c, and c’ depict the paths between the three variables, which are weighted by the regression coefficients. Significant coefficients (*p* < .05) are indicated with one asterisk. The total effect (path c) can be decomposed into two components, the direct effect and the indirect effect. Paths a and b represent the indirect effect that operates via the mediator, the number of training blocks on the contingency learning task. Path c’ represents the direct effect of JtC scores on the causal judgements, that is, the amount of predictive power left after the mediational effect of training length has been partialed out.

We described above an associative account of the causal illusion that could also explain why our measure of JtC correlates with the judgements of causality: as people high in the JtC bias would stop collecting information sooner in the contingency learning task, they should show a stronger pre-asymptotic illusion. To explore whether the length of training in the contingency learning task explains the correlation we have just reported, we performed a mediational analysis using bootstrapping procedures (see path model in Fig. 6). Effects and 95% confidence intervals were computed for 1000 bootstrapped samples.

We found a significant total effect (of JtC on causal illusion) but no evidence of a mediational structure: the direct effect was − 1.29, and the 95% confidence interval ranged from − 2.31 to − 0.39; whereas the indirect effect was − 0.03 with the 95% confidence interval ranging from − 0.24 to 0.16. Thus, we did find a relation between JtC and contingency training length in the contingency learning task as expected (see path a on Fig. 6) but the relation between the number of training trials and the judgements of causality was not significant (see path b on Fig. 6).

Overall, these results show that participants with higher tendency to jump to conclusions (measured as the amount of information gathered during the Beads Task) are also the ones with higher causal judgements on the contingency learning task, that is, those who developed higher illusions of causality. This means we have documented a correlation between the two cognitive biases, JtC and causal illusion. Then, we tested whether this correlation could be explained by the amount of training blocks in the contingency learning task, as associative models would predict. The mediational analyses did not support this causal chain in which causal judgements were modulated indirectly via the mediator variable, training length.

A potential limitation that may explain this null result is related to the sensitivity of our training length measure. Our participants were free to abandon the contingency learning task at the end of each 9-trials block. Nearly one half of the sample (forty-six participants) inspected only one block of trials in the contingency learning task, a similar proportion (forty-eight participants) inspected two blocks, and only six participants asked for three blocks. Thus, the training lengths that our participants received were in fact restricted to 9, 18, and 27 trials. This is a limited range that may have prevented us from finding a significant relationship between the training length and causal overestimations (path b). Additionally, when participants decided to gather more information, they were forced to observe a full new block, that is, nine additional medical records. This fixed amount of additional information may mask individual differences: participants who requested more trials might have preferred to observe fewer than nine additional medical records, if given the opportunity. Therefore, and in order to overcome this potential limitation, Study 2 will use a more sensitive measure of training length by allowing participants to stop gathering information on each trial, instead of at the end of each block.

## Study 2

The design of the study is similar to the previous one, but in order to improve the sensitivity of the measure of training length in the contingency learning task, we allow participants to stop collecting information on each trial, rather than waiting until the end of each block of trials. However, doing this may affect the judgements of causality because some parameters would be left uncontrolled. In particular, the critical parameters are the probability of the effect, the probability of the cue, and the actual contingency to which participants are exposed to^27^. In order to minimize the effect of these three factors, we used a fixed sequence of trials, identical for all participants.

### Method

#### Participants

Sixty-one Psychology undergraduate students (55 women and 6 men) volunteered for this study as an optional activity within a 90-min class session, and in return for course credits. We decided to use a sample of undergraduates who performed the task in a large computer room to avoid any effect of uncontrolled factors that might have been present in the online procedure of Study 1 (e.g., environmental conditions). Participants were aged between 18 and 25 years old (*M*_*age*_ = 18.87, *SD* = 1.41). The study was initially offered to a number of potential participants similar to the sample size in Study 1 (i.e., about 100). However, only a fraction of them eventually agreed to send their data to participate in the study. The effective sample size of 61 still allows for detecting effects of *r* = 0.30 or smaller with 80% power. We did not interrupt the data collection until the class session finished (therefore, the decision to stop collecting data was planned beforehand and could not be affected by the results).

### Instruments and apparatus

The task was presented as a web application based on World Wide Web Consortium (W3C) standards (i.e., HTML, CSS, and JavaScript) using the Google Chrome browser (a demo of this task can de download from the OSF).

### Procedure and design

The study was conducted simultaneously in two large computer rooms, in quiet conditions. Participants were verbally informed that all the data collected during the study will be sent anonymously to the researcher only upon explicit permission by the participant, indicated by clicking on a "Send" button that should appear at the end of the study. If the participant clicked on the "Do not send" button, the local information was erased. Participants could also choose their preferred language (Spanish or English).

**Figure 7:**
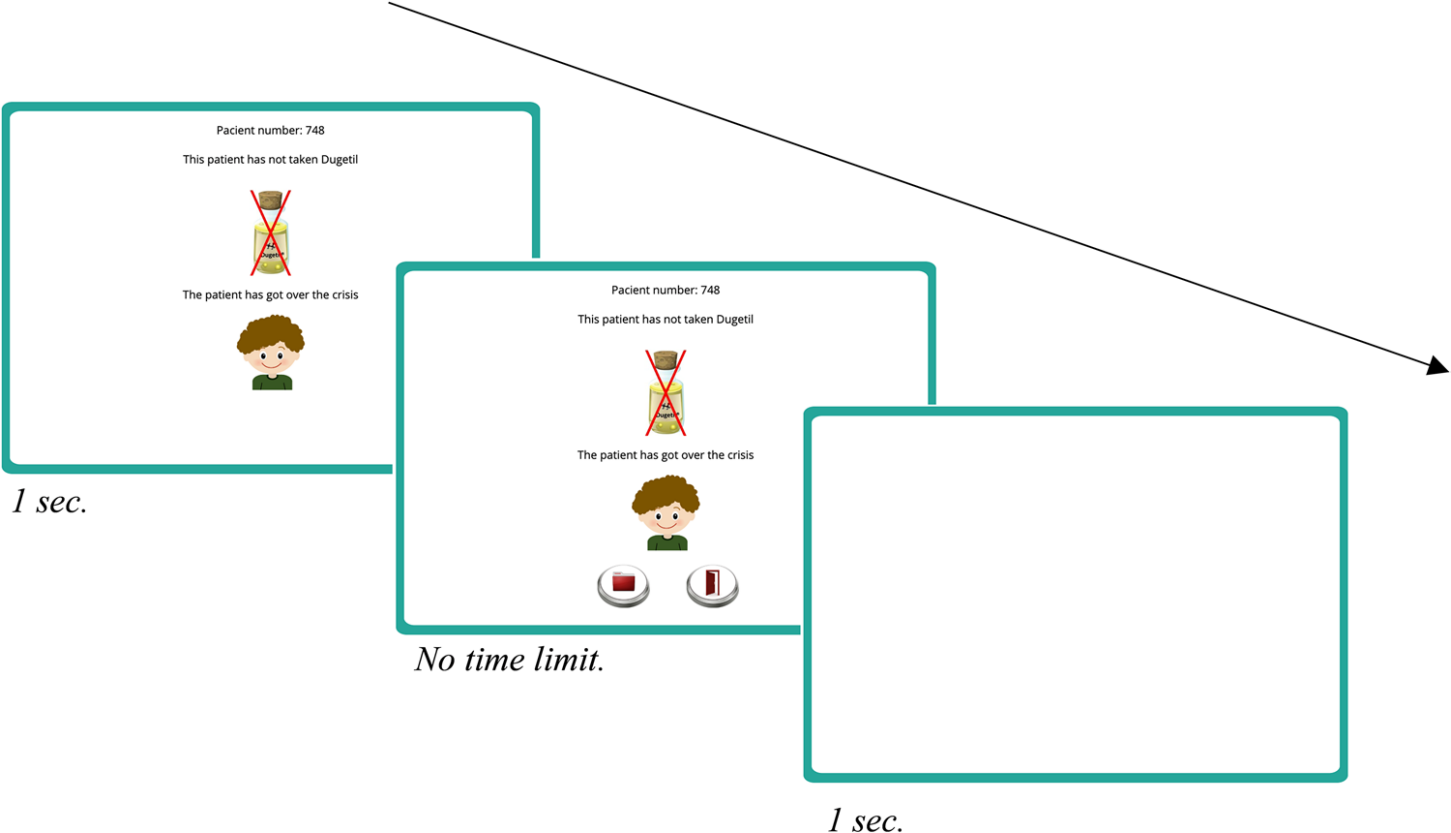
Schematic procedure of the contingency learning task used in Study 2 (this figure has been made up by combining actual screenshots from the original task).

We used the same version of the Beads task previously described for Study 1, but the contingency learning task was slightly different from that used in Study 1 (see detailed instructions in Supplementary Appendix A). Each trial started by presenting the patient number, the treatment and the outcome all together at the same time. Then, participants were required to choose between checking additional records, or judging the effectiveness of the medicine. The predictive question was not included because the participant's attention was already ensured by asking them to make this choice on each trial (see Fig. 7).

Those participants who decided to continue inspecting more records were presented with a new trial, whereas those who decided to answer were presented with a confirmation question, similar to the one used in Study 1 after each block of trials. This procedure allows more sensitivity than that used in Study 1, as it uses a training length measure with higher resolution (trials instead of blocks). Finally, participants confirming their choice were required to use a 100-point scale to rate the effectiveness of the medicine, the same way as in Study 1.

As mentioned before, we use a unique sequence of trials, identical for all participants. It included a total of 45 trials arranged in 7 blocks of nine trials. Each block maintained the same number of type “a”, “b”, “c”, and “d” trials that we used on Study 1 (4, 2, 2, and 1 respectively) ensuring that after each nine trials, p(E) = p(C) = 0.67 and Δp = 0. The sequence was designed to maintain a high p(E), a high p(C), and almost zero contingency regardless of the trial in which training is abandoned, allowing only small deviations of these parameters (see details on Supplementary Appendix C).

### Results

**Figure 8:**
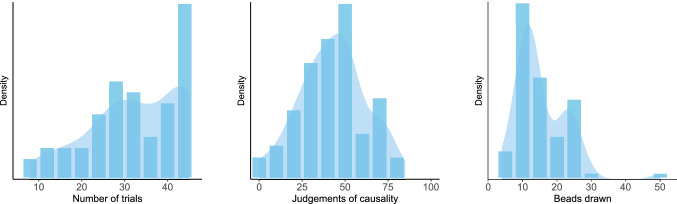
Histograms and density plots for the contingency learning task (left and central panels) and Beads task (right panel) in Study 2.

The number of training trials that participants chose to see in the contingency learning task ranged between 8 and 45 (*M* = 32.56, *SD* = 10.87, see left panel of Fig. 8). Causal judgements varied from 0 to 85 (*M* = 42.77, *SD* = 19.23, see central panel of Fig. 8), that is, although two participants (out of 61) noted that there was no relation between using the medicine and recovering from the crisis, most of them exhibited an illusion of causality and overestimated the effectiveness of the medicine, as we found too in Study 1. In the Beads task, the number of beads drawn before making a decision ranged between 1 and 50 (*M* = 15.48, *SD* = 7.67; see data distribution on the right panel of Fig. 8).

**Figure 9:**
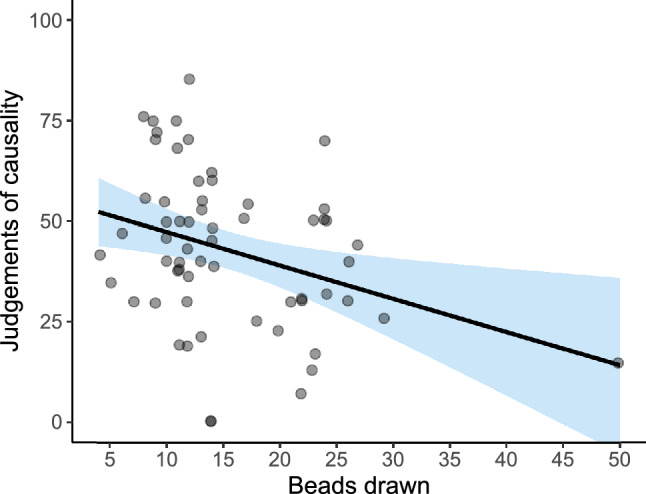
Scatterplot depicting judgements of causality as a function of JtC measure in Study 2. The scatterplot shows causal judgements (vertical axis), and the number of beads drawn in the Beads task (horizontal axis), which assesses the JtC bias. The line fitting the data points displays a negative slope, indicating that the participants who required more information in the Beads task were more accurate in their causal judgements. We added horizontal jitter to the data points to prevent overplotting.

Congruently with Study 1, we found a significant correlation between the number of beads drawn in the Beads task and the judgements of causality on the contingency learning task (see Fig. 9; *r* = -0.33, *p* = 0.009). That is, those participants with higher tendency to jump to conclusions were also the ones with stronger causal illusions.

**Figure 10:**
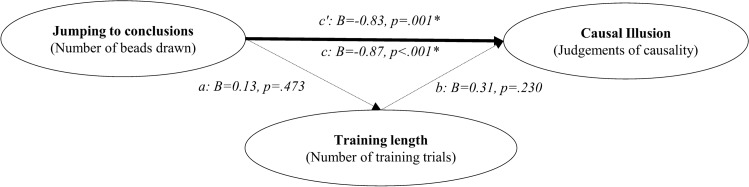
Path model in Study 2. The letters a, b, c, and c’ depict the paths between the three variables, which are weighted by the regression coefficients. Significant coefficients (*p* < .05) are denoted with an asterisk. The total effect (path c) can be decomposed into two components, the direct effect and the indirect effect. Paths a and b represent the indirect effect that operates via the mediator, the number of training blocks required in the contingency learning task. Path c’ represents the direct effect of JtC scores on the causal judgements, that is, the amount of predictive power left after the mediational effect of training length has been partialed out.

As in Study 1, we performed a mediational analysis to explore whether the training length in the contingency learning task can explain this relation, in line with the associative learning prediction (see path model in Fig. 10).

We found a significant total effect, but again no evidence of a mediational structure. The bootstrapped direct effect was − 0.83, and the 95% confidence interval ranged from − 1.37 to − 0.32; whereas the bootstrapped indirect effect was 0.04 with the 95% confidence interval ranging from − 0.08 to 0.24. Thus, it appears that JtC and causal illusions are associated in some way, but we failed to obtain evidence supporting the mechanism proposed by associative learning theories.

## Discussion

There is a well-supported conception that psychotic experiences may exist on a continuum with normal experiences^30,36^. This theoretical approach views psychosis as a phenotype of behaviours that can be examined across a continuum from general to clinical population. Among other factors, cognitive biases have been proposed to increase susceptibility to the development of abnormal beliefs, acting as a vulnerability factor that may contribute to experiencing clinical symptoms. Although some cognitive biases, such as Jumping to Conclusions (JtC), have been extensively explored in the context of delusional beliefs or at-risk mental states, as far as we know their contribution to non-clinical manifestations is less clear.

We carried out two studies in order to explore the potential implications of JtC on incorrect causal inferences (i.e., causal illusions) using samples from the general population (Internet users in Study 1, undergraduate students in Study 2). We expected that those participants with a higher tendency to jump to conclusions would display stronger causal illusions. Additionally, we proposed a causal mechanism based on associative learning theories to account for this relation: Those participants with high tendency to JtC are expected to gather a smaller amount of evidence before they reach a conclusion in the learning task, that is, they will need less information to decide whether the two events presented in this task are related or not. This reduced exposure to events may favour a pre-asymptotic learning effect, with causal inferences biased by the probability of the cause and the probability of the effect^39^. As predicted, we found a relationship between JtC and the magnitude of the causal illusions in both studies: The higher the tendency to jump to conclusions, the greater the overestimation in the contingency learning task. However, we did not find evidence supporting the above described associative mechanism either in Study 1 or in Study 2. That is, the effect appears not mediated by training length in the contingency learning task.

Our main result is the association between JTC and causal illusions, which was consistently found in both studies despite the procedural and sample differences between them. This suggests that the effect is general and can be replicated in a range of settings. However, the association found was not high either in Study 1 (*r* = − 0.28) or in Study 2 (*r* = − 0.33), suggesting a moderate contribution of the JtC bias to causal illusions in this type of scenario.

One possible explanation for the moderate size of the effect relates to procedural features of this research. For example, the contingency task is artificial and probably did not motivate participants to make accurate judgments. Thus, it is possible that a stronger contribution of JtC to causal estimations (or even a significant mediational role of training length) could be found in more ecological settings in which motivational components are allowed to play a role. Note that we cannot discard this possibility as our studies did not include a motivational component to specifically encourage participants to make accurate judgements. In fact, the weakest effect was detected on Study 1, an online study that compensated participants economically just for their participation, so they may not be motivated towards accuracy but rather to end the task as soon as possible (note that forty-six participants collected the minimum amount of information required to finish the task—one block of trials—).

An additional explanation for this moderate effect of JtC on causal illusions is related to the potential influence of uncontrolled demographic variables such as gender, education or age (note that the samples in the two studies may differ in these variables, which suggests some robustness of the findings despite variations in these factors). Previous research^14^ has provided no clear evidence that demographical variables affect data gathering behaviour after controlling for additional factors (i.e., cognitive style). Additionally, causal illusions seem to be general biases that appear regardless of gender, education, and age^27,55^. Nevertheless, future research could take into account these aspects to further describe the effect reported here and find boundary conditions that help us advance our understanding of the relationship between the two biases.

As mentioned above, the association between JTC and causal illusions was not mediated by training length in the contingency learning task, according to our analyses. We have already commented that motivational components may have contributed to this null effect, but one additional explanation is related to another procedural feature of our studies. Study 1 used a measure of training length that may lack sensitivity, as participants were forced to explore a fixed number of trials before being asked to draw a conclusion or to collect more evidence. Although this is a potential limitation for Study 1, the lack of sensitivity in this measure cannot explain the results in Study 2, because we allowed participants to stop information gathering at any trial in that study. Nevertheless, it is important to note that the increase of sensitivity in Study 2 comes with other limitations (i.e., less strict control of potentially relevant variables, and fixed trial sequence), as we will describe next.

As we noted at the beginning of this paper, causal judgements are known to be affected by factors like the probability of the potential cause or the probability of the effect. The fact that participants in Study 2 were allowed to abandon the training on any trial means that these parameters are in principle free to vary between individuals. We tried to control these deviations by using a fixed sequence of trials for all participants. Thus, unless participants collect an extremely small amount of information, the programmed sequence warrants that deviations are minimal (see Supplementary Appendix C). It is still true that, for those individuals stopping data gathering exceptionally soon (e.g., fewer than five trials), relevant parameters such as the p(O), p(C), and the actual contingency may had deviated from the programmed values. However, all participants asked for at least eight trials which means that deviations were actually negligible.

Apart from these limitations, it is also possible that the mechanism proposed (i.e., training length) was indeed not responsible for the relationship between JtC and causal illusions. Although this pre-asymptotic overestimation of causality is a clear prediction from associative learning theories, recent empirical research has shown that the amount of training may not produce a significant decrease in the intensity of causal illusions^75^. For example, Barberia et al.^75^ used a causal learning paradigm similar to the one that we have used for our studies, but with a fixed number of trials (to compare a short training phase to an unusually long training session). Their results showed that that causal illusions were not affected if training was increased from 48 up to 288 trials. In fact, they found moderate evidence against the hypothesis that extending the length of the training phase may reduce causal illusions, therefore suggesting that causal illusions may not be the consequence of incomplete learning. Thus, it is possible that a different mechanism accounts for the relation between JtC and causal illusions.

Fortunately, we can advance some ideas as to what kind of mechanism could underlie the effect, if it is not the pre-asymptotic bias. In addition to associative learning explanations, there is a second family of theories that have been used to account for contingency estimation and its deviations. These theories are based on statistical and probability rules (see Perales and Shanks' review on the different types of contingency learning models, associative and rule-based^76^). According to these theories, causal illusions may be explained as the result of uneven weighting of each type of evidence^77–79^. As some studies have shown, people do not give the same weight or importance to all four types of information in Fig. 1, which affects contingency estimations. This phenomenon can be captured in a weighted version of the Δp rule^80^, in which each type of evidence (“a”, “b”, “c”, “d”) has a weighting parameter (w):

1 $$ \Delta {\text{p}}_{{{\text{weighted}}}} = \left[ {{\text{w}}_{{\text{a}}} {\text{a}}/\left( {{\text{w}}_{{\text{a}}} {\text{a}} + {\text{w}}_{{\text{b}}} {\text{b}}} \right)} \right] - \left[ {{\text{w}}_{{\text{c}}} {\text{c}}/\left( {{\text{w}}_{{\text{c}}} {\text{c}} + {\text{w}}_{{\text{d}}} {\text{d}}} \right)} \right] $$

These weights (w) are free parameters reflecting relative differences in terms of attention or memory, and they endow the rule with the ability to predict systematic deviations in contingency estimation, without being subject to training length (unlike the associative explanation). In particular, previous literature has reported that type "a" events are weighted more heavily than other events (“a” > "b" = "c" > "d",^73,79^ but see^81^). Thus, if type "a" events are very frequent, which is the case of our studies, the contingency estimation is usually overestimated^77,79,82^. Note that this cell-weighting mechanism makes no prediction concerning the training length. That is, according to these theories, the causal illusion is not a pre-asymptotic effect.

In line with this account, previous research on psychosis has suggested that JtC may contribute to the onset and maintenance of delusions through a mechanism based on the differential salience of each type of evidence^59,83,84^. Specifically, Speechley et al.^84^ proposed that individuals with severe delusions give an extraordinarily high weight to the evidence that matches their hypotheses. The hypersalience of this evidence has been claimed to affect data gathering, making this process to finish prematurely when the initially received information aligns with the one that was expected. See Balzan et al.^59,85^ for compatible results with deluded and delusion prone individuals.

This mechanism, as described by Speechley et al.^84^, looks similar to the cell-weighting mechanism that was proposed in the contingency learning field. In fact, as we will describe next, the cell-weighting explanation could explain the relation between JtC and causal illusions that we reported in our studies. The instructions in the contingency learning task presented the medicine as a potential treatment for the crisis: “*The crises provoked by this disease could be healed immediately with a medication…”*. Consequently, we provided participants with the initial hypothesis that the medicine may heal patients suffering a crisis. With this hypothesis in mind, those individuals with higher sensitivity to the evidence that matches their hypothesis^84^ should give a higher weight to those pieces of evidence that align with this hypothesis, which in our task are “a” and “d” trials (i.e., trials in which the medicine is taken and the patient gets over the crisis, and trials in which the medicine is not taken and the patient does not get over the crisis). Hence, "a" and "d" trials would be given a higher weight than “c” and “b” trials. Taking into account our design (with a high proportion of type “a” trials) and this uneven appraisal of each type of information, we would expect that the more hypersalient to hypothesis-evidence matches a participant is (the more sensitive to information that confirms his/her hypothesis), the stronger his/her judgement about the relation between the medicine and the healings will be. This in turn may affect the data gathering process: as the very frequent, highly weighted, hypothesis-confirming trials accumulate (e.g., type "a" trials in which the cause and the effect are presented together), the confidence in the hypothesis increases rapidly, thus forcing the information sampling to finish early and leading to short training lengths.

Interestingly, the “evidence-hypothesis match” hypothesis aligns well with the literature on motivated reasoning^86,87^. This has been described as a process by which individuals will seek out information to support their own hypotheses (including weighting more heavily any piece of hypothesis-consistent information) and will down-weight information that does not support their hypotheses. Evidence for motivated reasoning has been described in the normal population^86,87^. Furthermore, previous research has proposed that motivated reasoning can modulate causal illusions in contingency learning tasks that are very similar to that in our studies, at least when the cover story is set in a highly motivational scenario (e.g., political decisions) in which participants were motivated towards giving a particular response, rather than towards being accurate^88^. Thus, it would not be surprising if motivated reasoning played a role in moderating the relation between JtC and causal illusion.

In particular, one of the ways in which this can happen is via a reduced motivation to be accurate in the task. First, it seems plausible that our participants were not motivated toward accuracy, and we argued that this could be an explanation for why the training length was not a significant mediator. This could be important, because individuals who are more motivated toward accuracy, and hence exhibit less of the motivated reasoning bias, may be more likely to complete a greater number of training trials. This means that only those participants who were motivated for accuracy and requested more training trails would eventually show the expected mediation. Consequently, future research could measure motivation as a potential moderator. Alternatively, motivation toward accuracy could be incentivized by means of monetary rewards or other procedures.

Additionally, another (related) motivational factor is the trait known as “Need for cognitive closure”^89^, which defines a motivation towards reducing uncertainty. It would be expected that those participants with greater need for closure would tend to form their beliefs more quickly, thus making decisions before collecting a big amount of information. This trait has been found associated to delusion-proneness^90^. However, previous studies failed to detect a direct link between Need for Closure and Jumping to Conclusions^90–92^. With respect to causal illusions, to the best of our knowledge, Need for Closure has not been studied as a potential factor contributing or modulating these biases. Thus, although it is not clear that this trait could play a role in explaining our current results, it could remain as a possible factor modulating causal illusions, and this could be investigated in future studies.

To sum up, the mechanism that clinical research has proposed to account for the relation between JtC and delusions parallels quite well the one proposed from the contingency learning research to describe how causal illusions are developed, and may also account for the relation that we found between these illusions and the tendency to stop data gathering hastily (JtC). From this perspective, individual differences could play a role in modulating the salience of each piece of information and, consequently, causal judgements and data gathering. Although we did not design our studies to test this possibility, our results are compatible with this mechanism.

## Conclusions

The results from this research showed an inverse relation between the tendency to jump to conclusions and causal illusions. We did not find evidence favouring an associative mechanism for this relation, which seems compatible with recent research on this topic suggesting that causal illusions are not pre-asymptotic effects^75^. Conversely, our results are compatible with a mechanism based on the hypersalience of the evidence-hypothesis matches^59,83,84^, which is similar to the cell-weighting mechanism proposed in the contingency learning literature. Further research should explore this second mechanism as a relevant individual marker for causal illusions in the general population. Such research should allow us to address the factors underlying abnormal beliefs and their contribution to the psychosis phenotype.

## Supplementary Information

Supplementary Information.

## Data Availability

The datasets generated during and/or analysed during the current studies are available in the Open Science Framework repository, https://osf.io/cbgrn.
