# Supplementary material for: The tendency to stop collecting information is linked to illusions of causality
Source: Sci Rep. 2021 Feb 16;11:3942. doi: 10.1038/s41598-021-82075-w (PMC7887230; doi:10.1038/s41598-021-82075-w)
Supplement: Supplementary file 1 — Supplementary Information. [file 41598_2021_82075_MOESM1_ESM.pdf]

The tendency to stop collecting information is linked to illusions of causality
María M. Moreno-Fernández, Fernando Blanco, and Helena Matute

Supplementary Appendix A

Instructions used for the contingency learning task in Experiment 1 and 2

Instructions used for Experiment 1

[Instructions, Screen 1]:

Imagine you are a Doctor who works in the Research Laboratory at a University. You are a Specialist in a very rare and dangerous disease called Lindsay Syndrome which requires urgent treatment in emergencies. The crisis provoked by this disease could be healed immediately with a medication called Batatrim, but this medication is still in experimental phase, so its effectiveness still hasn't been clearly checked.

[Instructions, Screen 2]:

Now, we're going to show you a series of medical records of patients who are suffering a crisis of the Lyndsay Syndrome. You'll see one patient per record and we'll tell you whether we have given them the Batatrim or not. We'll also tell you if the patient got over the crisis or not. Next you will observe the following patient. Try to find out if Batatrim is effective.

[New Block]:

"We still have medical records of patients with Lindsay Syndrome which we haven't shown to you. You can see some more records or let us know if Batatrim is effective to heal the Lyndsay Syndrome.
If you need to, we recommend you to see more records".
I want to see more patient records/I want to answer

[Training forced ending]

You have seen all the medical records we had of patients with Lyndsay Syndrome.
Now you should tell us if Batatrim is effective to heal the Lyndsay Syndrome.
Answer

[Judgement]

To what extent do you believe Batatrim is effective to heal the crisis of the Lyndsay Syndrome?
Non-effective/Quite effective /Totally effective
Selected value:
Click on the grey bar above and you'll see the cursor show up. You can drag this cursor to any point of the scale. Once you're happy with your answer, click the next button.

Instructions used for Experiment 2

[Instructions, Screen 1]:

Imagine you are a Doctor who works in the Research Laboratory at a University. You are a Specialist in a very rare and dangerous disease called Hankaoman Syndrome which requires urgent treatment in emergencies. The crises provoked by this disease could be healed immediately with a medication called Dugetil, but this medication is still in experimental phase, so its effectiveness still hasn't been clearly checked.

[Instructions, Screen 2]:

Now, we're going to show you a series of medical records of patients who are suffering a crisis of the Hankaoman Syndrome. You'll see one patient per record, and we'll tell you whether we have given them the Dugetil or not. We'll also tell you if the patient got over the crisis or not. Then, you will have to choose:

a) Either you can continue seeing more medical records in order to assess Dugetil's effectiveness.

b) Or, in case you are ready to give your response on Dugetil's effectiveness, you can stop seeing additional records and answer right away.

You will be allowed to check as many records as you want to find out if Dugetil is effective.

[Check before Judgement]:

"We still have medical records of patients with Hankaoman Syndrome which we haven't shown to you. You can see some more records or let us know if Dugetil is effective to heal the Hankaoman Syndrome.

If you need to, we recommend you to see more records.".

I want to see more patient records/I want to answer

[Training forced ending]

You have seen all the medical records we had. Now you should tell us if Dugetil is effective to heal the Hankaoman Syndrome.

Answer

[Judgement]

To what extent do you believe Dugetil is effective to heal the crisis of the crisis of Hankaoman syndrome?

Non-effective/Quite effective /Totally effective

Selected value:

Click on the grey bar above and you'll see the cursor show up. You can drag this cursor to any point of the scale. Once you're happy with your answer, click the next button.

Supplementary Appendix B

Instructions used for the beads task

[Instructions, Screen 1]:

In this part you are going to do a different task from the previous one. We have two containers with 100 beads. Both containers have red and blue beads, but in different proportion.

[Instructions, Screen 2]:

The container with the red lid has 60 red beads and 40 blue, this is to say, it has more red beads than blue.

[Instructions, Screen 3]:

However, the container with the blue lid only has 40 red beads and 60 blue, this is to say, it has more blue beads than red.

[Instructions, Screen 4]:

We have selected one of the two containers and we've poured its content in this card-board box. Your mission consists in finding out which one of the two containers we have selected. To achieve this goal, you'll be able to take out of the box as many beads as you want, until you have it clear which of the two containers we poured into the box, but you'll have to follow 3 rules…

[Instructions, Screen 5-7]:

1. You'll have to take the beads one by one.
2. Each time you take one bead out, you'll have to decide whether you want to take yet another bead out, or to tell us which of the two containers was poured into the box.
3. If you decide to see another bead, you'll have to place the one you have already removed back inside the box.

Supplementary Appendix C

Trial sequence used in the contingency learning task of Experiment 2

**Trial number**	**Trial type**	**Contingency** **Δp**	**Probability of the effect** **p(E)**	**Probability of the cause** **p(C)**
1	a	*Not computable*	1.00	1.00
2	c	0.00	1.00	0.50
3	c	0.00	1.00	0.33
4	d	0.33	0.75	0.25
5	b	-0.17	0.60	0.40
6	a	0.00	0.67	0.50
7	a	0.08	0.71	0.57
8	b	-0.07	0.63	0.63
**9**	**a**	**0.00**	**0.67**	**0.67**
10	a	0.05	0.70	0.70
11	c	-0.04	0.73	0.64
12	a	0.00	0.75	0.67
13	b	-0.08	0.69	0.69
14	d	0.07	0.64	0.64
15	b	0.00	0.60	0.67
16	a	0.04	0.63	0.69
17	c	-0.03	0.65	0.65
**18**	**a**	**0.00**	**0.67**	**0.67**
19	a	0.03	0.68	0.68
20	b	-0.02	0.65	0.70
21	a	0.00	0.67	0.71
22	a	0.02	0.68	0.73
23	b	-0.02	0.65	0.74
24	c	-0.07	0.67	0.71
25	d	0.02	0.64	0.68
26	c	-0.02	0.65	0.65
**27**	**a**	**0.00**	**0.67**	**0.67**
28	a	0.02	0.68	0.68
29	b	-0.02	0.66	0.69
30	d	0.05	0.63	0.67
31	a	0.07	0.65	0.68
32	c	0.03	0.66	0.66
33	c	0.00	0.67	0.64
34	a	0.02	0.68	0.65
35	b	-0.01	0.66	0.66
**36**	**a**	**0.00**	**0.67**	**0.67**
37	a	0.01	0.68	0.68
38	b	-0.01	0.66	0.68
39	c	-0.04	0.67	0.67
40	d	0.01	0.65	0.65
41	a	0.02	0.66	0.66
42	c	0.00	0.67	0.64
43	a	0.01	0.67	0.65
44	b	-0.01	0.66	0.66
**45**	**a**	**0.00**	**0.67**	**0.67**
